# Electroacupuncture Improves Pregnancy Outcomes in Rats with Thin Endometrium by Promoting the Expression of Pinopode-Related Molecules

**DOI:** 10.1155/2021/6658321

**Published:** 2021-04-15

**Authors:** Jin Xi, Jie Cheng, Chun-chun Jin, Jing-yu Liu, Zhen-ru Shen, Liang-jun Xia, Qian Li, Jie Shen, You-bing Xia, Bin Xu

**Affiliations:** ^1^Acupuncture and Tuina College, Nanjing University of Traditional Chinese Medicine, Nanjing 210023, China; ^2^Key Laboratory of Acupuncture and Medicine Research of Ministry of Education, Nanjing University of Traditional Chinese Medicine, Nanjing 210023, China; ^3^Xuzhou Medical University, Xuzhou 221004, China

## Abstract

A thin endometrium affects the success of assisted reproduction due to low endometrial receptivity. Acupuncture improves endometrial receptivity and promotes the formation of pinopodes, the ultrastructure marker implantation window. However, the specific underlying mechanisms remain unclear. In this study, the efficacy of acupuncture treatment and its underlying mechanism were investigated by analyzing pregnancy rate, pinopode formation, and related molecular markers in thin endometrium model rats. Absolute ethanol (95%) was injected into the uteruses of female Sprague-Dawley rats to construct a thin endometrium model. In this model, acupuncture stimulation at EX-CA1, SP6, and CV4 ameliorated the pregnancy rate. Significantly increased embryo implantation, endometrial thickness, numbers of glands, and blood vessels were observed in the electroacupuncture (EA) group compared to the model group. The number of pinopodes in the EA group was abundant, with a shape similar to that of the control group. Additionally, significantly higher expression levels of pinopode-related markers, including integrin *α*v*β*3, homeobox A10 (HOXA10), heparin-binding EGF-like growth factor (HBEGF), estrogen receptor alpha (ER*α*), and progesterone receptor (PR), were observed in the EA group than those in the model group. In conclusion, EA had a positive effect on the endometrial receptivity of thin endometrium model rats by improving pinopode formation through multiple molecular targets.

## 1. Introduction

Thin endometrium is often defined as an endometrial thickness below 7 or 8 mm on the day of ovulation or human chorionic gonadotropin administration [1–3]. Across published studies, the incidence of thin endometrium varies from 2.4% to 8.5% in patients undergoing assisted reproduction [4–6]. Possible pathological causes of endometrial thinning include dilatation and curettage of the uterus, intrauterine adhesions, infection, radiation, and endocrine dyscrasia [7].

Several interventions have been explored to improve endometrial thickness, such as luteal estradiol, granulocyte colony-stimulating factor (G-CSF), intrauterine infusion of platelet-rich plasma, and treatments that improve uterine blood flow [8]. Thus far, for patients with a thin endometrium, insufficient specific treatments have been clinically used to increase pregnancy or live birth rates [6]. Better therapies to treat thin endometrium are needed since it remains a frequent challenge in clinical contexts.

The success of assisted reproduction is regulated by the presence of a thin endometrium due to low endometrial receptivity [9]. The endometrial ability to accept embryos is defined as endometrial receptivity and is referred to as the “implantation window.” Pinopodes are the significant ultrastructural marker of the implantation window in both humans and rodents [10–14]. Fully developed pinopodes are closely related to the outcome of embryo implantation.

Acupuncture has been widely used as an effective nondrug therapy to treat female infertility in China for over 2000 years [15, 16]. The acupuncturist consultation rate regarding female infertility has increased significantly in the United Kingdom [17]. Based on certain improvements in endometrial receptivity, acupuncture promotes endometrial microcirculation and increases the clinical pregnancy rate of patients undergoing in vitro fertilization and embryo transfer [18–20]. Acupuncture also positively regulates various molecules related to endometrial receptivity through multiple targets and pathways [21–23].

A recent clinical trial showed that acupuncture increases the expression of endometrial pinopodes and improves endometrial receptivity [18]; however, the specific underlying mechanism remains unclear. Homeobox A10 (*HOXA10*) is a transcriptional regulatory gene expressed in the endometrium that plays a key role in embryo implantation [24]. A positive relationship has been observed between pinopodes and HOXA10 regulated by estrogen and progesterone at the genetic level [25, 26]. At the molecular level, various adhesion molecules and cytokines related to embryo implantation, such as integrin alpha v beta 3 (*α*v*β*3) and heparin-binding epidermal growth factor- (EGF-) like growth factor (HBEGF), are expressed on the surface of pinopodes [27, 28]. Clinical studies and animal experiments have shown that acupuncture increases the expression of Hoxa10 [29–31]. Similarly, acupuncture also regulates the expression levels of estrogen receptor alpha (ER*α*), progesterone receptor (PR), and integrin *α*v*β*3 to improve endometrial receptivity [30, 31].

In this study, synergistic changes in pinopodes and pinopode-related molecules were observed after acupuncture treatment in a thin endometrium rat model to preliminarily explore the mechanism and molecular targets of acupuncture regulating endometrial pinopodes.

## 2. Materials and Methods

### 2.1. Experimental Animals and Groups

Specific pathogen-free adult Sprague-Dawley rats (60 females and 20 males), aged 8-12 weeks, were purchased from Beijing Vital River Experimental Animal Technology and housed in the Animal Experimental Center of Nanjing University of Traditional Chinese Medicine. They were kept under a controlled 12 h light/dark cycle (lights on from 6 a.m. to 6 p.m. each day), and the temperature of the feeding environment was 22 ± 2°C with a relative humidity of 55 ± 10%. Standard diet and sterilized drinking water were supplied *ad libitum*. All animals were adapted to these conditions for 7 days before experimentation. This research was performed in accordance with the National Institutes of Health's Guide for the Care and Use of Laboratory Animals. All efforts were made to reduce animal suffering.

All female rats with normal estrous cycles were randomly divided into three groups according to the random number table method (*n* = 20 for each group): a control group, a model group, and an electro acupuncture group (EA group).

### 2.2. Model Establishment

The thin endometrium rat model was established as previously described [32, 33]. The right uterus was modeled during estrus. Anesthesia administration was performed using an isoflurane ventilator. A vertical incision (1–2 cm) was made 5 mm away from the right side of the ventrimeson on the lower abdomen. After exposing the abdominal cavity, the right uterus was gently pulled out. The junctions between the uterus, ovary, and proximal vagina were closed with two hemostatic clips. Approximately 0.3 mL of 95% absolute ethanol was slowly injected into the uterine cavity from the distal end of the ovary with a 1 mL syringe. After 5 min, the intrauterine ethanol was removed, and the clamps were removed. Then, the uterine cavity was flushed with sterile saline two to three times and the abdominal cavity was closed layer by layer. The pictures of experimental animals during modeling operation are listed in Supplemental materials (available here). The rats were kept warm after the operation and returned to the animal room after awakening.

### 2.3. EA Treatment

EA was performed 15 min per day after the day of modeling for three consecutive estrous cycles. Acupuncture needles (13 mm in length and 0.18 mm in diameter; Wuxi Jiajian Medical Devices Co., Ltd. Wuxi, China) were inserted to a depth of 5 mm at Sanyinjiao (SP6) and 2 mm at Zigong (EX-CA1) and Guanyuan (CV4). The depth and location of the EA, atlas of the skeleton, and anatomical locations were as described in *Experimental Acupuncture and Moxibustion* [34]. SP6 is located on the hind limbs, 10 mm above the tip of the medial malleolus. CV4 is situated 25 mm below the umbilicus of the rat abdomen. EX-CA1 is located on the abdomen, approximately 30 mm below the umbilicus, and 20 mm from the midline of the abdomen. Unilateral SP6 and EX-CA1 were connected to an EA treatment instrument with a positive electrode connected to SP6 and the negative electrode connected to EX-CA1, then alternating the next day. The stimulating intensity was 2 mA, and the frequency was 2/15 Hz.

### 2.4. Fertility Testing

Endometrial acceptance and retention of embryos were determined to evaluate endometrial receptivity. A vaginal smear was used to determine the estrous cycle of female rats. The female rats in each group were mated with sexually mature male rats in a 2 : 1 ratio on the day of estrus. The next morning, the presence of a vaginal plug was recorded at the beginning of pregnancy. Five random rats were selected from each group according to the random number table method to record the numbers and positions of embryo implantation on the eighth day of pregnancy. In the calculation of the number of implanted embryos, the number of embryos in the right uterus of each group was calculated.

### 2.5. Specimen Collection

The remaining twenty rats were euthanized by isolating the cervical spine under isoflurane anesthesia on the fifth day of pregnancy. The uterine tissue on the model side was harvested. The uteruses of five rats from each group were placed in 4% paraformaldehyde for H&E staining. The uteruses of five other rats from each group were frozen at -80°C for western blotting and quantitative reverse transcription polymerase chain reaction (RT-qPCR). The uteruses of the remaining rats were cut along the longitudinal axis, divided into blocks of approximately 2 mm^3^, and fixed in 2.5% glutaraldehyde for SEM.

### 2.6. H&E Staining

The uterus tissues were fixed in 4% paraformaldehyde at 4°C overnight, dehydrated in a graded series of ethanol, cleared in xylene, and embedded in paraffin. The isolated uterus tissues were sectioned into 5 *μ*m slides. One section of each tissue was routinely stained with H&E and viewed using an optical microscope (IX73; Olympus, Tokyo, Japan) by a group-blind researcher. Endometrial thickness and number of blood vessels and glands were measured using ImageJ software (National Institutes of Health (NIH), Bethesda, MD, USA). The endometrium thickness, comprising the vertical distance from the junction of the endometrium and myometrium to the uterine cavity, was recorded in four fields of each uterus section. The average of the four fields was used in the statistical analysis.

### 2.7. Scanning Electron Microscopy (SME)

The uteruses were dissected longitudinally and submerged in 2.5% glutaraldehyde for at least 24 h. After washing with phosphate-buffered saline three times and dehydrated with a gradient alcohol series (30%, 50%, 70%, 80%, 90%, and 100% ethanol), the samples were dried, mounted, and coated with gold. Finally, the rat endometrial surface was observed using SEM by a group-blind researcher (EVO-LS10; Zeiss, Oberkochen, Germany).

### 2.8. RNA Extraction and Reverse Transcription Quantitative-Polymerase Chain Reaction (RT-qPCR)

Total RNA was extracted on ice from the scraped endometrium using the TRIzol reagent and measured for concentration and purity using a spectrophotometer. Then, the reverse transcription reagent kit instructions were followed to construct the cDNA library and RT-qPCR was performed using the primers in Table 1 (Generay Biotech Co., Ltd., Shanghai, China) and PerfectStart Green qPCR SuperMix (IQ5TM, Bio-Rad, Hercules, CA, USA). The relative expression of mRNA was calculated using the 2^−*ΔΔ*Ct^ method.

### 2.9. Western Blotting

Total protein of the endometrium was extracted, and bicinchoninic acid was used to calculate the protein concentration. After denaturation at 100°C for 10 min, 10 *μ*g total protein was separated by sodium dodecyl sulfate-polyacrylamide gel electrophoresis and transferred to polyvinylidene difluoride membranes. The membranes were blocked with 5% bovine serum albumin for 2 h at room temperature and then incubated with primary antibodies overnight at 4°C with slow shaking. The primary antibodies used were as follows: anti-integrin alpha v beta 3 antibody (1 : 1000; Novus, USA), anti-HOXA10 antibody (1 : 1000; Abcam, UK), anti-HBEGF antibody (1 : 1500; Abcam, UK), anti-ER*α* antibody (1 : 1000; Abcam, UK), and anti-PR antibody (1 : 200; Abcam, UK). Anti-glyceraldehyde 3-phosphate dehydrogenase (GAPDH) antibody (1 : 1000; Cell Signaling Technology, Danvers, USA) was also added as an internal control. After washing with Tris-buffered saline with Tween, the membranes were incubated with anti-rabbit (1 : 5000; Abways, China) or anti-goat (1 : 10,000; Abbkine, China) IgG peroxidase antibodies. The protein was then exposed to a light-emitting liquid and imaged using a gel automatic imaging system (ChemiQ 4800mini; Bioshine, Shanghai, China). The gray value was measured with ImageJ software (NIH), and the relative expression was calculated.

### 2.10. Statistical Analysis

Data are presented as the mean ± standard error of the mean (SEM). One-way analysis of variance test was performed for compare the means of normally distributed parameters. The least significant difference test was used to calculate uniform variance, while Dunnett's T3 method was used to calculate uneven variance. Chi-square test and Fisher's exact test were used to calculate pregnancy rate. A value of *p* < 0.05 was considered statistically significant.

## 3. Results

### 3.1. Changes of Weight and Estrous Cycle in Thin Endometrium Model Rats

**Figure 1:**
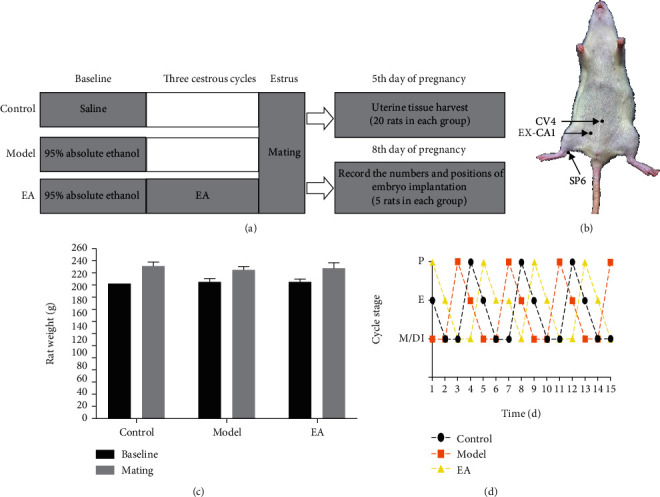
The experimental schema (a) exploring the effect of electroacupuncture (EA) and its underlying mechanism on thin endometrial model rats. (b) The acupoint location map shows the acupuncture treatment at acupoints CV4, EX-CA1, and SP6. (c) There is no significant difference in weight between the three groups, both at baseline and at mating. (d) Representative estrous cyclicity per group during the intervention period shows that all three groups of rats have a normal estrus cycle.

Following the schema (Figure 1(a)), the effect of EA on thin endometrial model rats was evaluated. Weight was recorded, and a vaginal smear was used to determine the estrous cycle of female rats every day. There were no significant differences in weight between the three groups at baseline (*p* > 0.05) and at mating (*p* > 0.05; Figure 1(c)). All three groups of rats had a normal estrus cycle (Figure 1(d)).

### 3.2. EA Increased Embryo Implantation in Thin Endometrium Model Rats

**Figure 2:**
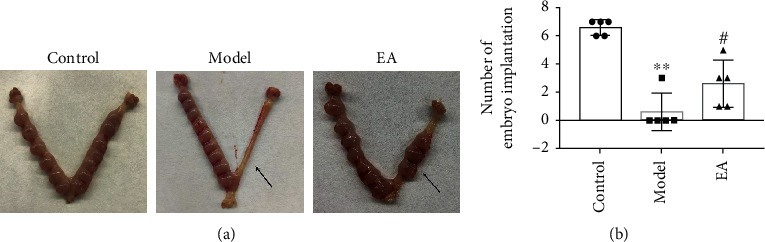
Electroacupuncture (EA) promotes embryo implantation in thin endometrial model rats. (a) The uterus specimen on the 8th day of pregnancy, with the arrow denoting the uterus after endometrial injury. (b) The number of embryo implantations in the injured uterus of the EA group is significantly increased compared to that in the model group. All data are expressed as the mean ± SEM (*n* = 5 per group). ^∗∗^*p* < 0.01 versus the control group; ^#^*p* < 0.05 versus the model group.

Rats on the 8th day of pregnancy were used to calculate the pregnancy rate and embryo implantation number. The number of embryo implantations was highest in the control group (*n* = 5), with 7, 6, 6, 7, and 7 embryos, respectively. In the model group (*n* = 5), there were no embryos in the injured uterus of four rats and the other rat had three embryos in the injured uterus. Significantly lower embryo implantation was identified in the model group than that of the control group (*p* < 0.01). As compared with the model group, the numbers of embryo implantations in the EA group were increased, which were 3, 5, 5, 3, 1, and 1 (*p* < 0.05; Figures 2(a) and 2(b)). The pregnancy rates of the control, model, and EA groups were 100%, 20%, and 100%, respectively. There were significant differences among the three groups (*p* < 0.05).

### 3.3. EA Repaired Endometrial Morphology during the Implantation Window

**Figure 3:**
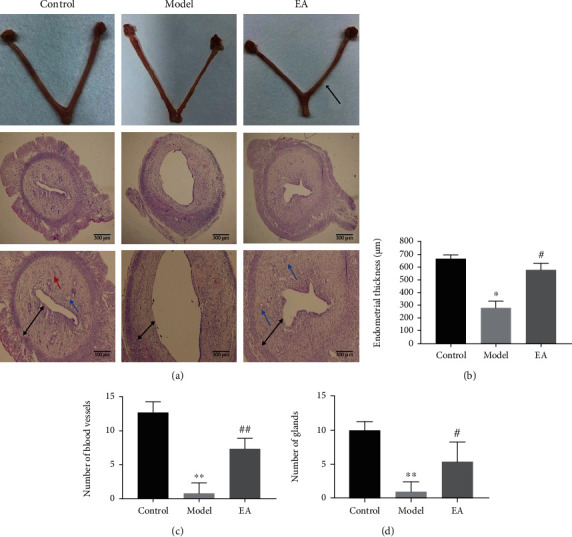
Electroacupuncture (EA) improves the morphology of the endometrium and promotes the formation of pinopodes. (a) The uterus specimen on the 5th day of pregnancy, with the arrow denoting the uterus after endometrial injury; Uterine tissue stained with hematoxylin and eosin (40x; 100x). Scale bar = 500 *μ*m. The red arrow points to the blood vessel, and the green arrow points to the gland. (b) Endometrial thickness in each group. (c) Endometrial gland numbers. (d) Endometrial blood vessel numbers. All data are expressed as the mean ± SEM (*n* = 5 per group). ^∗^*p* < 0.05 and ^∗∗^*p* < 0.01 versus the control group; ^#^*p* < 0.05 and ^##^*p* < 0.01 compared to the model group.

After modeling, the uterine lumens were thinner on the 5th day of pregnancy, while the lumens were thickened in the EA group and were similar to those in the control group (Figure 3(a)). In the model group, the uterine cavity was enlarged, the endometrium was significantly thinner, and its structure was incomplete with scarce numbers of glands and blood vessels, as determined by histology. In the EA group, a smaller uterine cavity, close packing in the endometrial basal layer, and increased numbers of blood vessels and glands were observed by histology (Figure 3(a)). A significantly thinner endometrium was observed in the model group than in the control group (*p* < 0.01; Figure 3(b)). In contrast, a significantly thicker endometrial lining was identified in the EA group than in the model group (*p* < 0.01; Figure 3(b)). Fewer glands and blood vessels were found in the model group than in the control group (*p* < 0.01, Figures 3(c) and 3(d)). However, significantly higher gland and blood vessel numbers were observed in the EA group than in the model group (*p* < 0.05, *p* < 0.01; Figures 3(c) and 3(d)).

### 3.4. Changes of Pinopode Expression and Its Related Molecules in Thin Endometrium Model Rats during the Implantation Window and the Ameliorative Effects of EA

**Figure 4:**
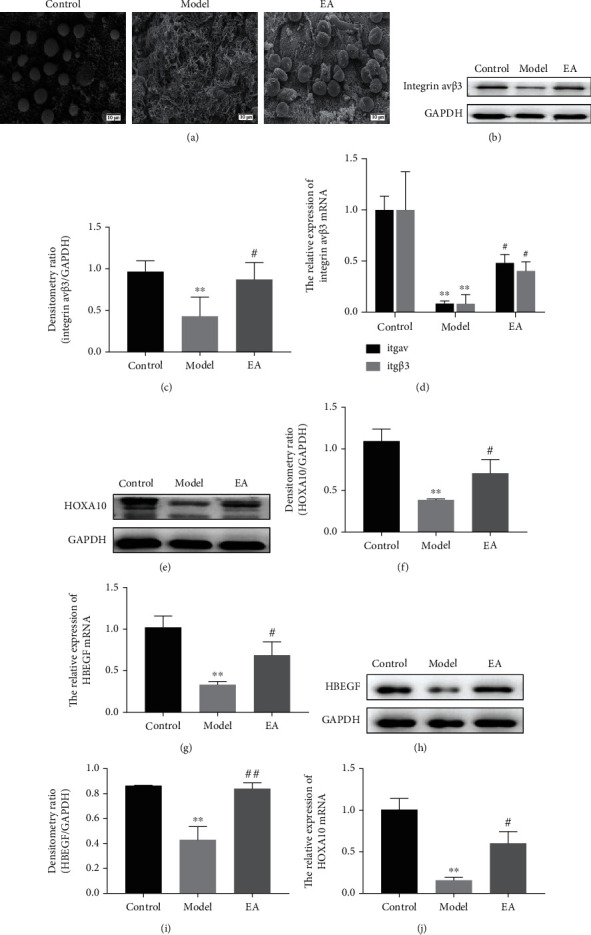
The pinopode and the expression of its related molecules in the endometrium are upregulated by electroacupuncture (EA) treatment in the thin endometrial rat model. (a) The endometrial pinopode is shown, as observed using scanning electron microscopy (SEM, 3000x). Scale bar = 10 *μ*m. (b) Western blot analysis of integrin *α*v*β*3 in the endometrium. (c) Relative expression levels of integrin av and integrin *β*3 mRNA. (d) Western blot analysis of HOXA10 in the endometrium. (e) Relative expression levels of HOXA10 mRNA. (f) Western blot analysis of HBEGF in the endometrium. (j) Relative expression levels of HBEGF mRNA. All data are expressed as the mean ± SEM (*n* = 5 per group). ^∗∗^*p* < 0.01 versus the control group; ^#^*p* < 0.05 and ^##^*p* < 0.01 versus the model group.

In the control group, the surface of the endometrium was flat with fully developed pinopodes on the microvilli, which were smooth and mushroom-like. In the model group, no pinopodes were observed in the endometrium. The number of pinopodes in the EA group was high, and they were well-developed, with a shape like that in the normal group (Figure 4(a)). To identify the effect of EA on pinopodes, the expression of its related molecules was explored, including integrin *α*v*β*3, HOXA10, and HBEGF, which are involved in embryo implantation. Protein and mRNA expression levels were assessed during the implantation window by performing western blotting and RT-qPCR, respectively.

Significantly decreased expression of integrin *α*v (*Itgav*) and integrin *β*3 (*Itgβ3*) mRNAs was identified in the model group compared with that in the control group (*p* < 0.01, *p* < 0.01; Figure 4(c)). However, after EA treatment, the mRNA expression levels of *Itgav* and *Itgβ3* were significantly upregulated (*p* < 0.05, *p* < 0.05; Figure 4(c)). The results of western blot analysis were consistent with the mRNA expression levels. A significantly higher expression of integrin *α*v*β*3 was observed in the EA group than in the model group (*p* < 0.05; Figure 4(b)). The protein and mRNA expression of HOXA10 was significantly downregulated in the model group compared with that in the control group (*p* < 0.01, *p* < 0.01; Figures 4(d) an(d) 4(e)). However, HOXA10 expression was significantly upregulated in the EA group relative to that in the model group (*p* < 0.05, *p* < 0.05; Figures 4(d) and 4(e)). Additionally, the protein and mRNA expression of HBEGF was significantly downregulated in the model group compared with that in the control group (*p* < 0.01, *p* < 0.01; Figures 4(f) and 4(j)). However, the protein and mRNA expression of HBEGF was significantly upregulated in the EA group relative to that in the model group (*p* < 0.01; *p* < 0.05; Figures 4(f) and 4(j)).

### 3.5. Changes of ER*α* and PR Expression in Thin Endometrium Model Rats during the Implantation Window and the Ameliorative Effects of EA

**Figure 5:**
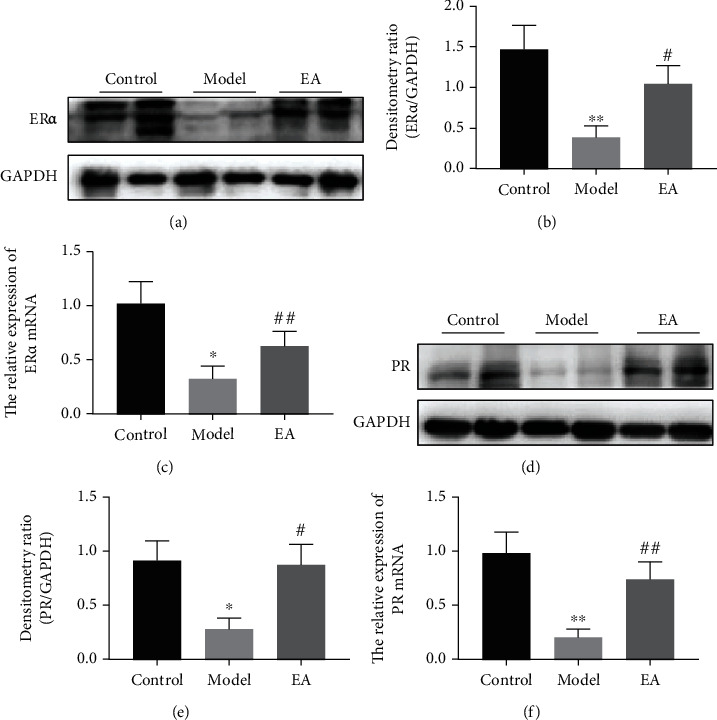
The expression of estrogen receptor alpha (ER*α*) and progesterone receptor (PR) in the endometrium is upregulated by electroacupuncture (EA) treatment in the thin endometrial rat model. (a) Western blot analysis of ER*α* in the endometrium. (c) Relative expression levels of ER*α* mRNA. (d) Western blot analysis of PR in the endometrium. (e) Relative expression levels of PR mRNA. All data are expressed as the mean ± SEM (*n* = 5 per group). ^∗^*p* < 0.05 and ^∗∗^*p* < 0.01 versus the control group; ^##^*p* < 0.01 versus the model group.

The protein and mRNA expressions of ER*α* were significantly downregulated in the model group relative to those in the control group (*p* < 0.01, *p* < 0.05; Figures 5(a) and 5(b)). However, the protein and mRNA expressions of ER*α* were significantly upregulated in the EA group compared with those in the model group (*p* < 0.05, *p* < 0.01; Figures 5(a) and 5(b)). Significantly decreased PR expression was identified in the model group compared with that in the control group (*p* < 0.05, *p* < 0.01; Figures 5(c) and 5(d)). However, after EA treatment, PR was significantly upregulated compared to that of the model group (*p* < 0.05, *p* < 0.01; Figures 5(c) and 5(d)).

## 4. Discussion

Embryo implantation failure caused by the thin endometrium is a serious challenge for assisted reproductive technology, especially since there is no established effective treatment for this condition. In recent years, long-term administration of estrogen, vaginal sildenafil, low-dose aspirin, pentoxifylline, autologous platelet-rich plasma, intrauterine perfusion of G-CSF, and other treatments have been used to treat thin endometrium; however, providing effective evidence-based interventions continues to pose a significant challenge [8, 35–37]. Therefore, alternative treatments are needed for patients with thin endometrium. Acupuncture has been widely used around the world, and its positive effect on endometrial receptivity has been confirmed in many studies [20]. However, the efficacy of acupuncture on thin endometrium and its mechanism remain unclear.

The injection of 95% ethanol caused severe injurie to the uterus, leading to disruption of the endometrial structure, scarce numbers of endometrial glands and blood vessels, and a thin endometrium. Treatment with EA significantly reduced endometrial injury. After EA treatment, the endometrial morphology was improved, the endometrium thickened, and the number of blood vessels and glands increased.

The embryo implantation rate is an important parameter for evaluating fertility in female rats. In this research, it was demonstrated that, compared with the control group, the embryo implantation rate of the model group was significantly reduced. Furthermore, a significant increase in the embryo implantation rate of rats that received EA treatment was observed, indicating that EA improved pregnancy outcome.

In this study, both endometrium regeneration and endometrial receptivity after EA were assessed. Endometrial receptivity refers to the ability of the endometrium to receive embryos; it provides an opportunity for the embryo to attach, invade, and develop, eventually forming a new individual and continuing the species [38]. Embryo implantation in rats occurred on the fifth day of pregnancy. Thus, the fifth day of pregnancy was chosen to study the expression of endometrial receptivity markers to evaluate endometrial receptivity more accurately [39].

Several clinical studies suggest that endometrial epithelial cell pinopode expression is the best morphological marker to evaluate endometrial receptivity [40, 41]. The mature pinopode only exists in the implantation window period, which occurs 6-8 days after ovulation and lasts for 48 h [42]. The timing of the appearance, maturation, and degeneration of the pinopode almost precisely coincides with the opening and closing of the endometrium “implantation window,” which is used to predict the stage of embryo implantation [40]. Mature pinopodes contain essential receptors and pregnancy-related proteins, which promote the smooth process of embryo adhesion and implantation [43]. Compared with other parts of the endometrium, the membranous protrusion of the pinopode greatly increases the contact area between the embryo and the endometrium. Therefore, the endometrium where the pinopodes are located adheres strongly to the embryo, and the embryo always implants where there are pinopodes. In the rat endometrium, the number of pinopodes has been found to increase on the fourth day of pregnancy, peak on the fifth day, and rapidly decline on the sixth day [14, 44]. Here, it was found that endometrial pinopodes were abundant and well-developed during the implantation window in the EA group. Therefore, EA promoted the formation of pinopodes to increase the embryo implantation rate of thin endometrium model rats.

The expression of several pinopode biomarkers was explored, including integrin *α*v*β*3, HOXA10, and HBEGF, which also play key roles in embryo implantation. In this study, changes in these molecules after acupuncture were evaluated to clarify the acupuncture target molecules for pinopode regulation.

Integrins are a family of heterodimeric transmembrane receptors that mediate cell adhesion, which is the key step in embryo implantation [45]. A large number of animal experiments and clinical trials have confirmed that integrin *α*v*β*3 is closely related to endometrial receptivity, and its expression is significantly low in endometria with unexplained infertility [46–49]. As a potential receptor for blastocyst attachment, integrin *α*v*β*3 is located on the pinopode. Furthermore, the appearance of pinopodes is closely associated with increased levels of integrin *β*3 [50–52].

As a transcription factor, HOXA10 plays an important role in uterine receptivity during implantation, functions as a key regulator of endometrial decasualization, and is regulated by ovarian steroid hormones [53]. Downregulation of HOXA10 results in a reduced number of pinopodes, while overexpression of HOXA10 has the opposite effect [25]. In addition, integrin *α*v*β*3 and pinopode implantation efficiency are suppressed by HOXA10 methylation [54].

HBEGF, a member of the epidermal growth factor family, is essential for successful implantation [55]. It binds to its specific receptor on trophoblastic cells to mediate embryo adhesion to the endometrium and ameliorate oxidative stress-mediated uterine decidualization damage [56, 57]. *In vitro* experiments have demonstrated that HBEGF promotes the expression of HOXA10 and integrin *α*v*β*3 in epithelial cells [58]. Another study has shown that HBEGF is a transmembrane molecule expressed on the surface of fully developed pinopodes that are present on the luminal and glandular epithelium [26].

This research revealed higher expression levels of integrin *ανβ*3, HOXA10, and HBEGF after EA treatment, indicating that EA improved endometrial receptivity and might play a role in regulating molecules associated with pinopodes to promote their formation.

Uterine function is regulated by estrogen and progesterone, which modulate gene expression levels of the luminal and glandular epithelium, as well as stromal cells [59]. The physiological effects of estrogen and progesterone are mediated by their receptors. ER*α* appears to be essential for implantation since ER*α*-knockout mice have shown endometrial hypoplasia and infertility [60]. Similarly, the adult female PR mutant displays significant defects in all reproductive tissues [61]. After progesterone and estrogen binding, their receptors activate a series of signal transduction pathways, including regulating the expression levels of HOXA10 and HBEGF [62–64]. The response of endometrial stromal cells to progesterone depends on the regulation of HOXA10 during embryo implantation and decasualization, as HOXA10 is present in uterine stromal cells in a PR-dependent manner [65]. A genomic study identified HOXA10 as a critical target of PR in endometrial stromal cells [66].

**Figure 6:**
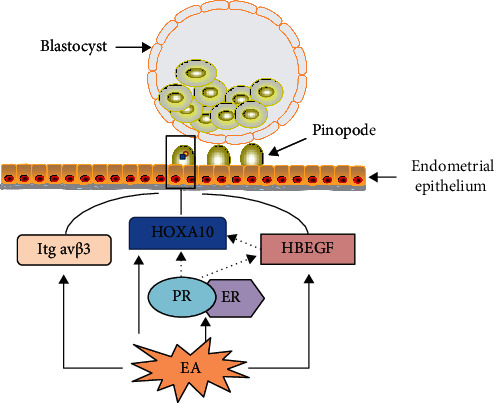
The mechanism of electroacupuncture (EA) improves embryo implantation by regulating the expression of pinopode-related molecules. EA has a positive effect on the endometrial receptivity of thin endometrium model rats by improving pinopode formation through multiple molecular targets. After EA treatment in thin endometrial model rats, the embryo implantation rate is improved, endometrial structure is repaired, the formation of pinopodes on the endometrium is increased, and the expression of pinopode-related molecules, including integrin *α*v*β*3, HOXA10, HBEGF, ER*α*, and PR, is upregulated. In addition, regulation of ER*α* and PR may affect the expression of HOXA10 and HBEGF.

In this research, the expression levels of ER*α* and PR were explored at the transcription and translation levels. Both ER*α* and PR were upregulated after EA treatment compared to those in the model group. Therefore, the regulation of ER*α* and PR, which affect the expression levels of HOXA10 and HBEGF, may be one of the mechanisms of EA to improve the formation of pinopodes in thin endometrium model rats (Figure 6).

As far as we know, this research is the first to explore the effect of EA on the formation of endometrial pinopodes in thin endometrial model rats with promising results. However, there are limitations to this study. First, this study did not observe litter size, which is an important parameter for assessing reproduction in female rats. Second, the mechanism of EA treatment on thin endometrium was not explored in depth. Third, the present study only evaluated the therapeutic effects of EA and did not observe the efficacy of other drug treatments or combination therapies. In clinical applications, the combined use of acupuncture and other treatment methods may be more effective. This study suggested that EA might be considered as a potential treatment for thin endometrium. However, further studies on the mechanism of EA treatment of thin endometrium are needed.

## 5. Conclusions

In conclusion, this study demonstrated that acupuncture had a positive effect on the endometrial receptivity of thin endometrium model rats by improving the formation of pinopodes through multiple molecular targets, including integrin *α*v*β*3, HOXA10, HBEGF, ER*α*, and PR.

## Figures and Tables

**Table 1:** Gene sequences for primer synthesis.

Gene name	Forward (5′>3′)	Reverse (5′>3′)
Itgb3	TCGTTGATGCTTACGGGAAAA	TGGGATGACCTCGTTGTTGAG
Itgav	TTGGCTGCCGTTGAGATAAGA	CCACTGGAGGTTCAGGATTGC
Hoxa10	TTACACGAAGCACCAGACG	TCCTGCGATTCTGAAACCAG
Hbegf	AGACATTTCCCTTATCCTGCC	CGCCAACCTTCTCTGTACTAAG
Pgr	TGTCATTCTACTCGCTGTGC	ACCTCATCTCTTCAAACTGGC
Esr1	AGATGGTCAGTGCCTTATTGG	AGATTCAAGTCCCCAAAGCC
Actin	CCCATCTATGAGGGTTACGC	TTTAATGTCACGCACGATTTC

## Data Availability

The initial data used to support the findings of this study are available from the corresponding author upon request.

## Supplementary Materials

Supplementary Materials Supplementary materials are the pictures of experimental animals during modeling operation. Supplementary figure 1: the position of the abdominal incision: a vertical incision (1–2 cm) was made 5 mm away from the right side of the ventrimeson on the lower abdomen. Supplementary figures 2 and 3: the uterus when 95% absolute ethanol was injected into the uterine cavity and removed.
