# Supplementary material for: Electroacupuncture Improves Pregnancy Outcomes in Rats with Thin Endometrium by Promoting the Expression of Pinopode-Related Molecules
Source: Biomed Res Int. 2021 Apr 15;2021:6658321. doi: 10.1155/2021/6658321 (PMC8062184; doi:10.1155/2021/6658321)
Supplement: Supplementary Materials — are the pictures of experimental animals during modeling operation. Supplementary figure 1: the position of the abdominal incision: a vertical incision (1–2 cm) was made 5 mm away from the right side of the ventrimeson on the lower abdomen. Supplementary figures 2 and 3: the uterus when 95% absolute ethanol was injected into the uterine cavity and removed. [file 6658321.f1.zip › The concise description for supplementary (1).docx]

Supplementary materials are the pictures of experimental animals during modeling operation. Supplementary figure 1 shows the position of the abdominal incision: a vertical incision (1–2 cm) was made 5 mm away from the right side of the ventrimeson on the lower abdomen. Supplementary figure 2 and 3 show the uterus when 95% absolute ethanol was injected into the uterine cavity and removed.
