# Supplementary figures and images for: Electroacupuncture Improves Pregnancy Outcomes in Rats with Thin Endometrium by Promoting the Expression of Pinopode-Related Molecules
Source: Biomed Res Int. 2021 Apr 15;2021:6658321. doi: 10.1155/2021/6658321 (PMC8062184; doi:10.1155/2021/6658321)

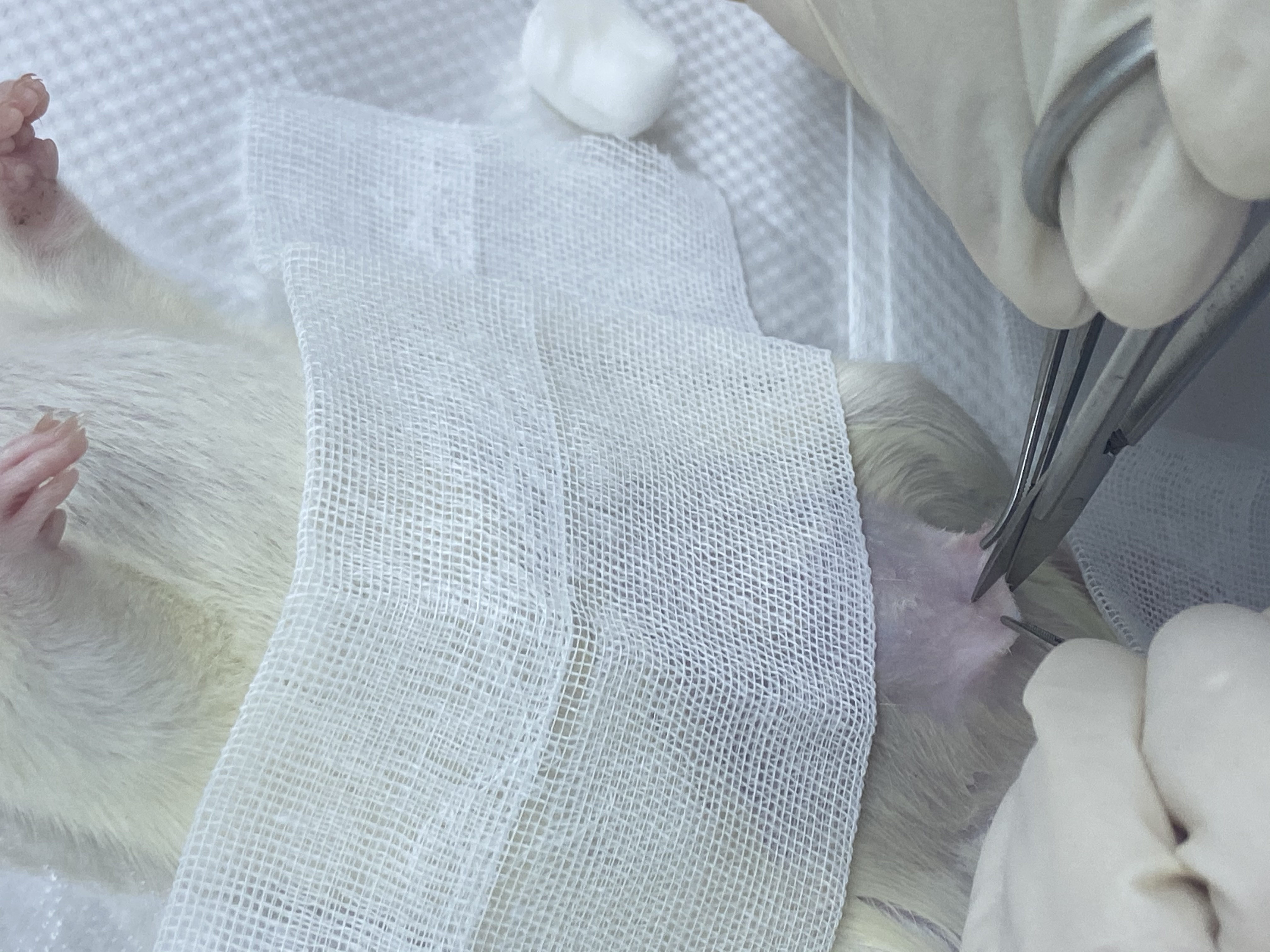

Supplement: Supplementary Materials — are the pictures of experimental animals during modeling operation. Supplementary figure 1: the position of the abdominal incision: a vertical incision (1–2 cm) was made 5 mm away from the right side of the ventrimeson on the lower abdomen. Supplementary figures 2 and 3: the uterus when 95% absolute ethanol was injected into the uterine cavity and removed. [file 6658321.f1.zip › 1.The position of the abdominal incision.jpg]

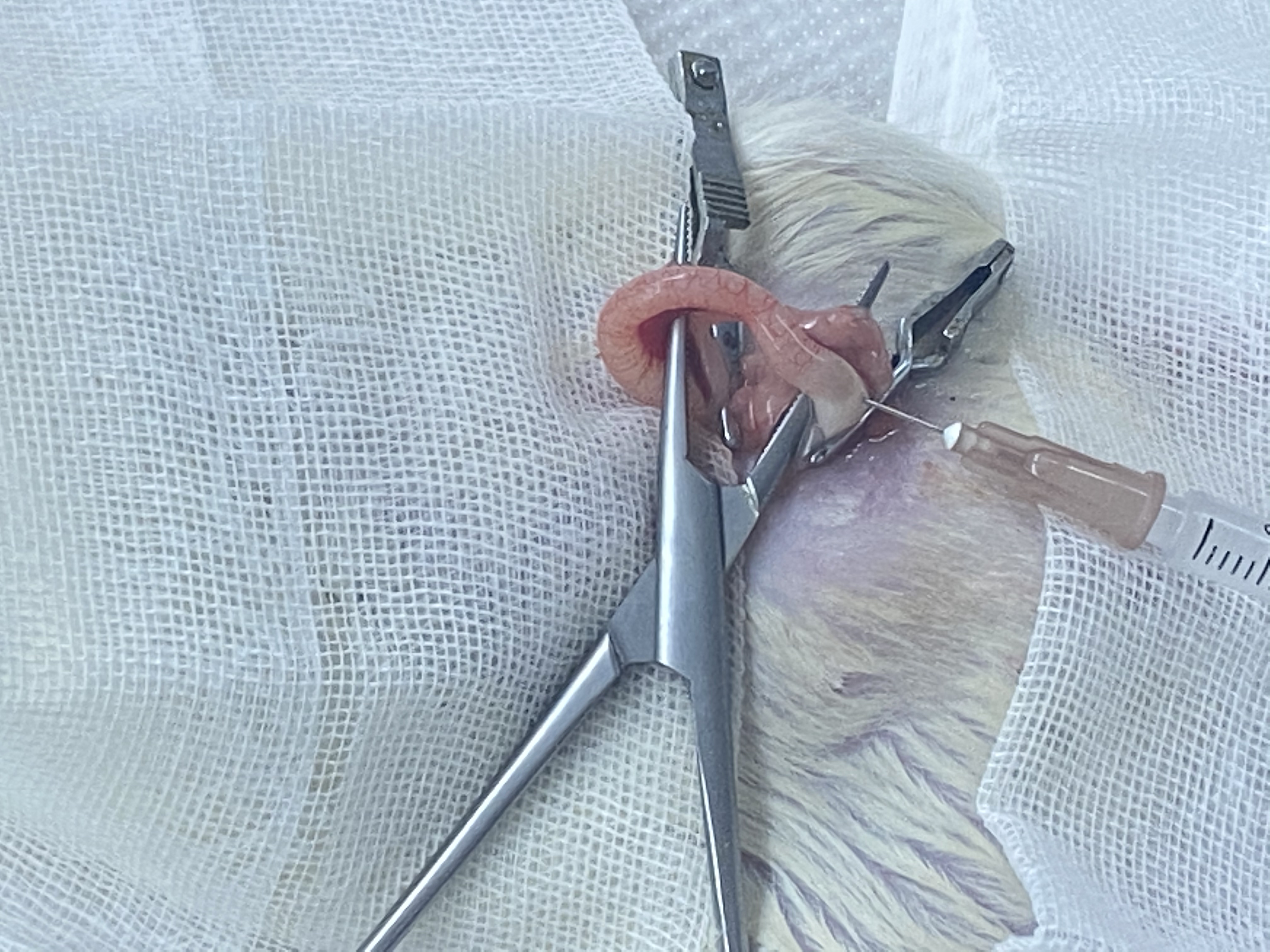

Supplement: Supplementary Materials — are the pictures of experimental animals during modeling operation. Supplementary figure 1: the position of the abdominal incision: a vertical incision (1–2 cm) was made 5 mm away from the right side of the ventrimeson on the lower abdomen. Supplementary figures 2 and 3: the uterus when 95% absolute ethanol was injected into the uterine cavity and removed. [file 6658321.f1.zip › 2.Uterine perfusion with absolute ethanol.jpg]

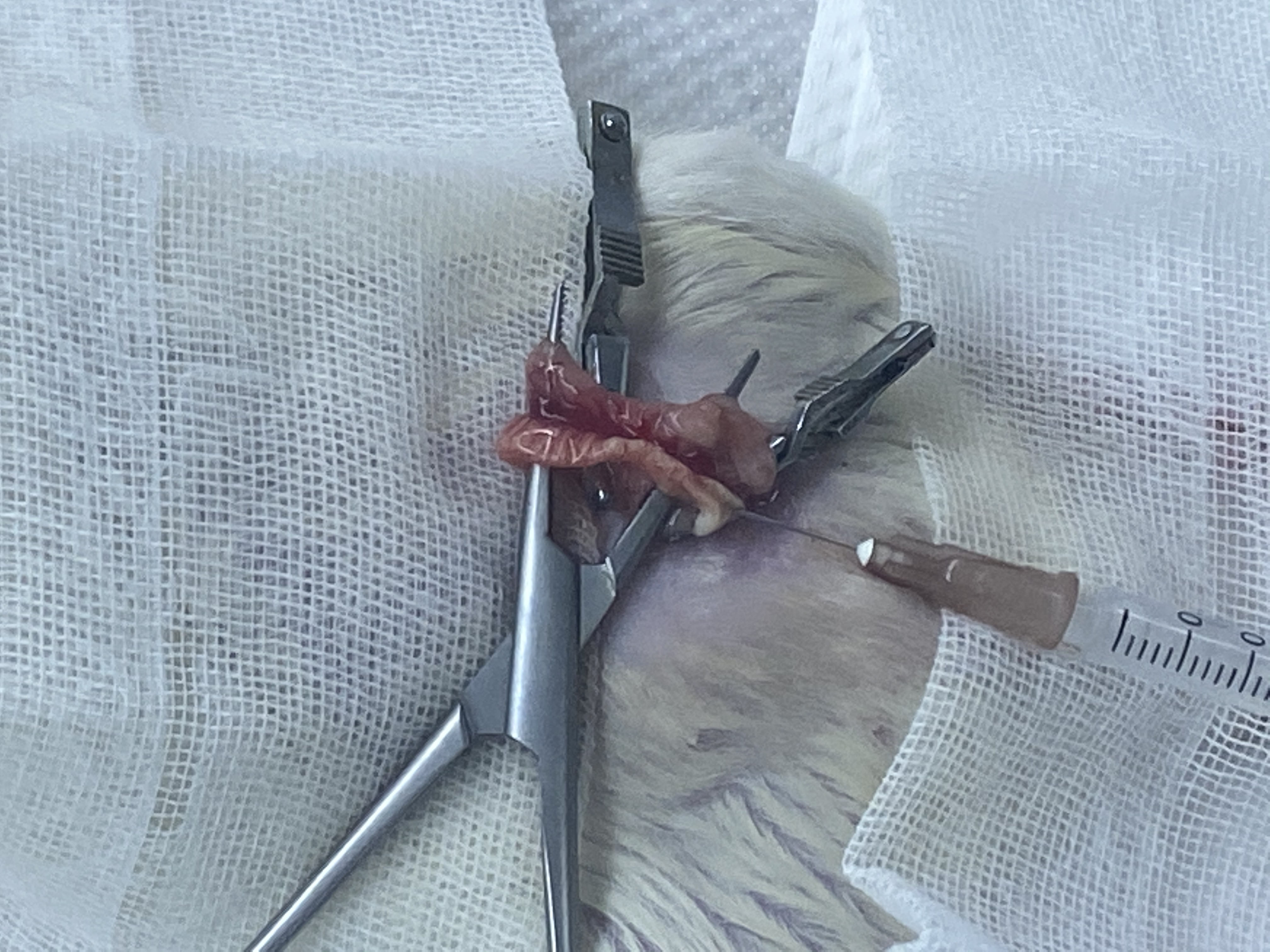

Supplement: Supplementary Materials — are the pictures of experimental animals during modeling operation. Supplementary figure 1: the position of the abdominal incision: a vertical incision (1–2 cm) was made 5 mm away from the right side of the ventrimeson on the lower abdomen. Supplementary figures 2 and 3: the uterus when 95% absolute ethanol was injected into the uterine cavity and removed. [file 6658321.f1.zip › 3.The intrauterine ethanol was removed.jpg]
